# Epidemiological profile of early childhood caries in a sub-urban population in Nigeria

**DOI:** 10.1186/s12903-021-01780-0

**Published:** 2021-08-23

**Authors:** Morenike Oluwatoyin Folayan, Ayodeji Babatunde Oginni, Maha El Tantawi, Tracy L. Finlayson, Abiola Adeniyi

**Affiliations:** 1 Department of Child Dental Health, Obafemi Awolowo University, Ile-Ife, Nigeria; 2 Innovative Aid, Abuja, Nigeria; 3 Department of Pediatric Dentistry and Dental Public Health, Faculty of Dentistry, Alexandria University, Alexandria, Egypt; 4 San Diego State University, San Diego, USA; 5 Department of Preventive Dentistry, Lagos State University College of Medicine, Ikeja, Lagos State Nigeria

**Keywords:** Early childhood caries, Dmft, Dmfs, Pufa, ICDAS, Nigeria

## Abstract

**Background:**

The aim of the study was to determine the prevalence and severity of early childhood caries (ECC) in children 6–71-months; identify the teeth most at risk for ECC; and identify risk indicators associated with significant caries index (SiC) score in different age groups.

**Methods:**

This was a cross-sectional study that collected data (using a household survey) on the ECC risk indicators (frequency of tooth brushing, consumption of refined carbohydrate in-between-meals, daily use of fluoridated toothpaste, and dental service utilization in the 12 months) in Ile-Ife, Nigeria. We computed the prevalence of ECC using the International Caries Detection and Assessment System (ICDASI (d^1–6^)) index; caries severity using the ICDAS-2(d^1–2^) and ICDAS-3(d^3–6^) for non-cavitated and cavitated lesions respectively, decayed missing, filled teeth (dmft), and surfaces (dmfs) and SiC indices; and caries complications using the pulp (p), ulceration (u), fistula (f) and abscesses (a) (pufa) index, for children 6–11-months-old, 12–23-months-old, 23–35-months-old, 35–47-months-old; 48–59-months-old and 60–71-months-old. The differences in the mean dmft, dmfs, pufa scores, and ICDAS 1, 2, and 3 scores, and proportion of children with each ECC risk indicator were computed. Logistic regression analysis was conducted to identify risk indicators for the ECC SiC index score for each age group.

**Results:**

The prevalence of ECC was 4.7%: 2.9% had non-cavitated lesions and 2.8% had cavitated lesions. The mean (SD) dmft, dmfs and pufa scores were 0.13 (0.92), 0.24 (1.91) and 0.04 (0.46) respectively. The dmft and dmfs scores were highest among the 24–35-months-olds while the SiC score was highest among the 12–23-months-olds. There were no significant differences in dmft, dmfs, and pufa scores between the different age groups. Toothbrushing more than once a day was the only factor associated with the SiC score: it decreases the odds for the SiC score in children 48–59-months-old. The teeth worst affected by ECC were #85 and #61.

**Conclusion:**

The prevalence, severity and risk indicator for ECC seems to differ for each age group. The granular details on the risk profile of children with ECC in this population with a low ECC prevalence and burden can allow for the planning of age-targeted interventions.

## Background

Early childhood caries (ECC) is a disease of public health importance. Caries in primary teeth are the tenth most prevalent disease in children [1]. When left untreated, it has a significant negative impact on the growth and development of the affected child [2, 3] and reduces the quality of life of both the child and the caregiver [4]. Caries epidemiological studies help monitor the prevalence of disease and identify associated risk indicators among different age and geographical groups, thus providing new treatment perspectives [5]. The caries profile of a population of young children can be summarized and compared using different indices thereby harnessing the advantages of the indices to derive a comprehensive and relevant epidemiological profile. Several indices are available for assessing dental caries at the population level, each offering various benefits and associated challenges.

An index commonly used to characterize dental caries experience at the tooth level is the decayed (d), missing due to caries (m), and filled (f) teeth (dmft) index. Another similar index is the decayed (d), missing due to caries (m) and filled (f) surfaces (dmfs) index for primary teeth [6]. These indices yield information about the prevalence and severity of caries in an individual, and they are frequently used as a clinical measure for oral health assessment in national surveys [7]. The scores for dmft range from 0 to 20; and the dmfs index scores range from 0 to 88. A score of zero indicates no caries, while scores greater than zero indicate the presence of caries, with increasing scores indicating greater severity. The indices had been used for macro-level assessment of the impact of governance on the oral health of children [8]. These indices have remained in use for over 80 years, despite known limitations including an equal weight given to decayed teeth and well-restored teeth; each tooth or tooth surface can be counted only once. Also, it does not provide a valid indication of treatment need and does not account for the number of teeth or surfaces at risk [9–11].

The International Caries Detection and Assessment System (ICDAS) is another established index that enables the detection of non-cavitated and cavitated enamel and dentine caries [12]. Its advantage over the dmfs/dmft scoring is its ability to facilitate the detection of non-cavitated lesions, especially enamel caries [13]. ICDAS helps develop a treatment plan [14] thereby overcoming one of the limitations of the dmft/dmfs score [15]. ICDAS also has good reproducibility and validity to detect caries lesions and estimate their severity though it seems to overestimate caries activity of cavitated lesions [16]. It also has the disadvantage of long application time when compared with the dmft/dmfs indices [17].

The pufa index assesses complications associated with caries which may be more serious than the carious lesions themselves. The presence of teeth with open pulp (p), ulceration (u), fistula (f), and abscesses (a) indicate the severity of the complications associated with a carious tooth [18]. The index is simple to use [19] and has been validated for use in Nigeria [20]. For a country like Nigeria where the prevalence of untreated caries is high [21], the pufa index can provide useful information for policy formation and oral health care planning.

The significant caries index (SiC) is computed based on the mean dmft of one-third of the sample with the highest caries experience scores [22, 23]. It is useful for identifying variations in decay proportions alongside the dmft [24], especially in countries where the overall decay experience is low [24, 25]. SiC resolves the problem related to skewed caries distribution when using the dmft/dmfs. Its use, however, needs to be complemented with other relevant information to enhance planning for caries management [26].

Overall, despite the limitations of the identified indices, they are invaluable for population-level caries management. We provide an age-specific report on ECC experience in children 6–71-months-old resident in a semi-urban area of Nigeria using these indices thereby harnessing their potential to develop an epidemiological profile of ECC in Nigeria.

Past studies on the profile of ECC in Nigeria had reported on the prevalence [27, 28] and risk factors for ECC in different communities in the country [27–30]. Caries are affected by multiple factors including the caries risk behaviors of individuals. Caries risk behaviors include the regularity of toothbrushing, frequency of consumption of cariogenic diet, use of fluoridated toothpaste, and the use of preventive dental services for preventive dental care [31]. None of these studies conducted to date had reported on age group-specific risk indicators of ECC in Nigeria. A more granular detail on the severity of ECC measured using the dmft/dmfs, ICDAS, pufa, and the SiC indices will enhance the planning for specific age group intervention. We also report on age-specific ECC risk indicators of the study population.

## Methods

The data for this study was extracted from that of a primary cross-sectional study conducted to determine the association between maternal psychological factors and ECC in children 6–71 months old. Clinical examination and household survey data were collected between December 2018 and January 2019 in the Ile-Ife local government area of Osun State, Nigeria.

The methodology for the survey had been described in prior publications [32–36]. The primary study required a minimum sample of 1439 computed by using a prevalence of 6.6%, a margin of error of 5%, and a confidence level of 95%. The sample was recruited from households distributed proportionately across the 11 political wards. In each political ward, enumeration centers were randomly selected from the north, south, east, west and central axis of each ward to obtain samples representative of the local government area. A total of 70 of the 700 enumeration centres in the local government areas were randomly selected. At each of the enumeration sites, every other household on each street was eligible for recruitment of the child. A child was recruited from each household. Children recruited from each household were less than 6 years of age, living with a caregiver, present at the time of the survey, had at least one tooth erupted into the mouth, and with caregiver’s consent to participate in the study. In any household where more than one child was eligible to participate, the study participant was selected by simple ballot. Whenever a household declined to participate, it was substituted with the next eligible household. Children who had chronic medical conditions requiring prolonged use of sweetened medications and those with medical conditions that increased their risk of ECC were excluded from the study.

The oral examinations were conducted by five calibrated dentists using the dmft, dmfts, pufa, and ICDAS indices. The Kappa statistic for intra-examiner agreement ranged from 0.80 to 0.92. The inter-examiner agreement between each of the dentists and the trainer ranged from 0.80 to 0.89. The inter-examiner agreement for pairs of the five dentists ranged from 0.82 to 0.91. The children were examined either sitting on a chair or their mother’s lap. Illumination was through the use of natural daylight without the use of a magnifying lens and air drying. Visual examinations were performed with plane dental mirrors. No radiographs were used.

### dmf indices

The dmf index was determined using the World Health Organization criteria at the cavitation level [7]. The dmft score was obtained by adding the d, m, and f scores for each child at the tooth level while the dmfs index was the sum of the d, m, and f scores at the tooth surface level.

### The pufa index

The ‘p’ indicated pulpal involvement when the opening of the pulp chamber was visible or when coronal structures had been destroyed by caries and only roots or root fragments were left. No probing was used to diagnose pulpal involvement. The ‘u’ indicated ulceration due to trauma from sharp pieces of tooth and was recorded when sharp edges of a dislocated tooth with pulpal involvement or root fragments caused traumatic ulceration of surrounding soft tissues, e.g. tongue or buccal mucosa. The ‘f’ indicated fistula and was scored when a pus-releasing sinus tract related to a tooth with pulpal involvement was present. The ‘a’ indicated abscess and was scored when a pus containing swelling related to a tooth with pulpal involvement was present. To arrive at a pufa score, the number of carious primary teeth with pulpal involvement, carious teeth with ulceration, and carious teeth with fistula and with abscess was determined and summed for each child [18].

### The ICDAS index

Caries status was assessed with the ICDAS II index [31]. The ICDAS II scores for dental caries diagnosis included (0) Sound tooth; (1) First visual change in enamel; (2) Distinct visual change in enamel; (3) Localized enamel breakdown; (4) Underlying dark shadow from dentine; (5) Distinct cavity with visible dentine; (6) Extensive distinct cavity with visible dentine [37]. The severity of ECC was measured with ICDAS II using three different thresholds: ICDAS-1, identifying any caries lesion at any stage (d^1–6^); ICDAS-2, including non-cavitated lesions (d^1–2^), and ICDAS -3: including advanced lesions (d^3–6^). In place of compressed air, cotton rolls were used to dry the tooth surfaces like was done in a prior study outside the clinic [38]. No radiographs were taken. The teeth were examined after removing dental plaque from tooth surfaces and dried using gauze pads. The mesial, distal, buccal, lingual, and occlusal surfaces (where present) were examined.

### SiC index

The SiC Index is the mean dmft of one-third of the study group with the highest caries score. To calculate the SiC index for each age group, we sorted each age group according to their dmft, selected one-third of the children in each age group with the highest dmft, and calculated the mean dmft for per age group [39].

### ECC risk indicators

The ECC risk indicators of each child were assessed using the tool developed by Khami et al. [31] and adapted for use with children, adolescents, and young people in Nigeria [36, 38, 40, 41]. Information was generated on the frequency of tooth brushing, consumption of refined carbohydrate in-between meals, daily use of fluoridated toothpaste, and dental service utilization in the 12 months preceding the study. Information was also obtained about a child’s sex and age in years which was classified for analysis into one-year age bins.

Respondents were asked to indicate the frequency of consuming sugar-containing snacks or drinks in-between meals using the following options—‘about three times a day or more, ‘about twice a day’, ‘about once a day’, ‘occasionally’, ‘not every day’, ‘rarely’, or ‘never between meals’. Responses were dichotomized into ‘three times a day or more’ and ‘less than three times a day’. Consumption of refined carbohydrates (sugar containing snacks or drinks) in-between-meals three times a day or more was classified as an ECC risk indicator [28, 36, 42].

Respondents were also asked to indicate the time of their last dental check-up as follows —within the last 6 months, more than 6 months to one year ago, more than 1–2 years ago, more than 2–5 years ago, more than 5 years, never, do not remember. Respondents who chose the options within the last 6 months and more than 6 months to one year ago were classified as having undertaken ECC preventive dental care while those who chose the other options were classified as having ECC risk indicator [38, 43].

Respondents were asked to indicate the frequency of their use of fluoridated toothpaste when tooth brushing using the following options—‘always’, ‘quite often’, ‘seldom’, ‘not at all’. Respondents who answered ‘always, were classified as having undertaken ECC preventive dental care and all others were classified as having ECC risk indicator [38].

Respondents were also asked to indicate the frequency of tooth brushing using the following response options–‘irregularly or never’, ‘once a week’, ‘a few (2–3) times a week’, ‘once a day’, and ‘more than once a day’. Responses were further dichotomized into ‘more than once a day’, considered good ECC preventive practice versus ‘once a day or less’ which was considered an ECC risk indicator [38, 44].

### Data analysis

Age was categorised into 6–11-months-old, 12–23-months-old, 23–35-months-old, 35–47-months old; 48–59-months-old and 60–71-months old. The mean dmft, dmfs, and pufa scores for each age group and each tooth type were computed. The prevalence of ECC was calculated as the proportion of participants with ICDAS-1(d^1–6^) greater than zero. The percentage of children with ECC was determined according to two ICDAS II thresholds (ICDAS-2(d^1–2^), and ICDAS-3(d^3–6^)) for non-cavitated and cavitated carious lesions respectively. The differences in mean dmft, dmfs, pufa scores, and the percentages of children with ICDAS 1, 2, and 3 scores among age groups were computed using ANOVA and Chi test respectively.

The prevalence of each ECC risk indicator—frequency of tooth brushing, consumption of refined carbohydrate in-between-meals, daily use of fluoridated toothpaste, and dental service utilization in the 12 months preceding the study—was computed for each age group. The differences in the prevalence of the ECC risk indicators among age groups were also compared using the Chi test.

Linear regression analysis was done to identify risk indicators for the ECC SiC index. Statistical analyses were conducted with Stata/SE 14.0 for Windows. The significance level was set at *p* < 0.05.

## Results

Table 1 provides an overview of the ECC profile of the 1549 study participants. ECC prevalence using ICDAS (d^1–6^) was 4.7%–73 of the 1549 children examined had ECC: 45 (2.9%) had non-cavitated lesions (ICDAS d^1−2^) and 43 (2.8%) had cavitated lesions (ICDAS d^3−6^). There was no significant difference in the prevalence of non-cavitated (ICDAS d^1−2^) (*p* = 0.137) and cavitated (ICDAS d^3−6^) (*p* = 0.204) lesions among the 6 age groups.

**Table 1:** ICDAS dmft, dmfs, pufa and SiC index scores by age of children 6–71-months-old in Ile-Ife, Nigeria (N = 1549)

Age	No. of children examined n (%)	ICDAS 0 n (%)	ICDAS 1–2 n (%)	ICDAS 3–6 n (%)	ICDAS 1–6 n (%)	Mean (SD) dmft	Mean (SD) dmfs	Mean (SD) pufa	SiC index
6–11 months	125 (8.1)	123 (98.4)	1 (0.8)	1 (0.8)	2 (1.6)	0.03 (0.36)	0.11 (0.89)	0.0 (0.0)	4
12–23 months	244 (15.8)	237 (97.1)	5 (2.1)	3 (1.2)	7 (2.9)	0.12 (1.16)	0.29 (3.24)	0.05 (0.77)	6
24–35 months	262 (16.9)	246 (93.9)	14 (5.3)	6 (2.3)	16 (6.1)	0.27 (1.54)	0.36 (2.53)	0.04 (0.50)	4
36–47 months	355 (22.9)	340 (55.8)	9 (2.5)	11 (3.1)	15 (4.2)	0.14 (0.80)	0.22 (1.43)	0.05 (0.47)	3
48–59 months	335 (21.6)	315 (94.0)	9 (2.7)	14 (4.2)	20 (6.0)	0.10 (0.47)	0.20 (0.96)	0.04 (0.29)	2
60-71 months	228 (14.7)	215 (94.3)	7 (3.1)	8 (3.5)	13 (5.7)	0.07 (0.45)	0.21 (1.12)	0.02 (0.21)	2
Total	1549 (100.0)	1476 (95.3)	45 (2.9)	43 (2.8)	73 (4.7)	0.13 (0.92)	0.24 (1.91)	0.04 (0.46)	3
*p*-values		0.18¶	0.14¶	0.20¶	0.18¶	0.12¥	0.846¥	0.89¥	

¶Chi-square, ¥ ANOVA

The mean (SD) dmft for all age groups was 0.13 (0.92) and the mean (SD) dmfs was 0.24 (1.91). The dmft and dmfs scores were highest among the 24–35-months-old age group. There were no significant differences in the mean dmft (*p* = 0.115) and mean dmfs (*p* = 0.846) scores among the different age groups.

The mean (SD) pufa score was 0.04 (0.46) with scores ranging from 0 in 6–11-months-olds to 0.05 among 12–23-months-olds and 36–47-months-old children. Though there was no age-related pattern observed in ECC related complications as reflected by the pufa scores, we noticed less severity in the age 48–59-months-old group to the 60–71-months-old group. There was no significant difference in the severity of complications among the age groups (*p* = 0.891).

The SiC score also increased from 4 in 6–11-months-old to 6 in 12–23-months-old and thereafter, the score decreased to 4 in 24–35-months old to 2 in 48–59-months-old and 60–71-months old respectively. The SiC score was higher than the group mean score in children 6–47-months-old.

Table 2 highlights the details of the dmft, dmfs and pufa for each tooth. The teeth with the topmost mean (SD) dmft scores were #51 (0.014 (0.118)) followed by #85, #61, #52 (0.012 (0.11), and #84, #62 (0.011 (0.104)). The lower canines and the lower incisors had the least scores (#73, #53, #81, #63, #72, #83, and #82 respectively). The teeth with the highest mean (SD) dmfs scores were #85 and #61 followed by #54, #84, #74 and #64. The lower left and upper right canines, and the lower central incisors have the least scores (#73, #53, #81, #71 respectively). The teeth with the highest pufa scores were #64, #85, #61 and #75; while the upper canines and lower central incisors did not have complications though they had carious lesions.

**Table 2:** The mean and standard deviation of the dmft, dmfs, and pufa scores by tooth type in 6–11-months-old children in Ile-Ife, Nigeria (N = 1549)

Tooth	Dmft		Dmfs		Pufa	
	Mean	SD	Mean	SD	Mean	SD
51	0.014	0.118	0.018	0.168	0.001	0.025
52	0.012	0.110	0.016	0.188	0.003	0.051
53	0.002	0.044	0.003	0.062	0.000	0.000
54	0.009	0.095	0.021	0.236	0.003	0.051
55	0.004	0.062	0.009	0.172	0.003	0.051
61	0.012	0.110	0.023	0.237	0.005	0.111
62	0.011	0.104	0.015	0.160	0.001	0.036
63	0.002	0.044	0.005	0.116	0.000	0.000
64	0.008	0.088	0.019	0.243	0.006	0.134
65	0.005	0.067	0.008	0.129	0.001	0.025
71	0.003	0.051	0.003	0.067	0.000	0.000
72	0.002	0.044	0.005	0.098	0.000	0.000
73	0.001	0.036	0.001	0.025	0.001	0.025
74	0.008	0.091	0.019	0.239	0.002	0.044
75	0.010	0.098	0.018	0.218	0.004	0.062
81	0.002	0.044	0.003	0.062	0.000	0.000
82	0.002	0.044	0.007	0.141	0.001	0.025
83	0.002	0.044	0.007	0.167	0.001	0.025
84	0.011	0.104	0.019	0.218	0.003	0.051
85	0.012	0.110	0.023	0.225	0.005	0.114
Total	0.131	0.920	0.241	1.901	0.037	0.458

Table 3 shows the age-specific distribution of ECC risk indicators in the study participants. The proportion of children who consumed refined carbohydrate in-between-meals three times a day or more, utilized the dental service in the 12 months preceding the study, used fluoridated toothpaste daily, and brushed their teeth more than once a day was 18.9%, 5.8%, 90.8%, and 93.2% respectively. Significantly fewer children consumed refined carbohydrate in-between-meals three times a day or more among those aged 6–11-months-old than other age groups (*p* < 0.001). Also, significantly fewer children used fluoridated toothpaste among those aged 6–11-months-old than other age groups (*p* < 0.001). There was no significant difference in the proportion of children who utilized the dental service in the 12 months preceding the study (*p* = 0.482) and the proportion of children who brushed their teeth more than once a day (*p* = 0.251) by age group.

**Table 3:** Proportion of children 6–71-months-old children in Ile-Ife, Nigeria with caries risk behaviors by age group (N = 1549)

Age	Intake of refined carbohydrate in-between-meals three times a day or more n (%)	Dental service utilization in the past 12 months n (%)	Use of fluoridated toothpaste n (%)	Toothbrushing more than once a day n (%)
6–11	8 (6.4)	8 (6.4)	79 (63.2)	118 (94.4)
12–23	32 (13.1)	13 (5.3)	218 (89.3)	228 (93.4)
24–35	40 (15.3)	15 (5.7)	233 (88.9)	236 (90.1)
36–47	47 (13.2)	28 (7.9)	338 (95.2)	331 (93.2)
48–59	41 (12.2)	16 (4.8)	322 (96.1)	319 (95.2)
60–71	43 (18.9)	10 (4.4)	217 (95.2)	211 (92.5)
Total	211 (18.9)	90 (5.8)	1407 (90.8)	1443 (93.2)
p value	< 0.001	0.482	< 0.001	0.251

Table 4 shows the risk indicators for the SiC score in the different age groups. Sex, intake of refined carbohydrate in-between-meals three times a day, use of fluoridated toothpaste, and dental service utilization were not significantly associated with SiC scores in any of the six age groups. The only significant risk indicator was tooth brushing more than once a day. Children aged 48–59 months who brushed their tooth more than once a day had significantly lower SiC score when compared to children brushed their tooth once a day or more (B = 0.11; 95% CI 0.02, 0.61).

**Table 4:** Logistic regression analysis for caries risk behaviors associated with ECC for 6–71-months-old children in Ile-Ife, Nigeria with caries risk behaviors by age group (N = 1549)

Age	SiC index					
	6–11 months	12–23 months	24–35 months	36–37 months	48–59 months	60–71 months
	AOR (95% CI)	AOR (95% CI)	AOR (95% CI)	AOR (95% CI)	AOR (95% CI)	AOR (95% CI)
	*p* value	*p* value	*p* value	*p* value	*p* value	*p* value
Sex						
Male			1.00	1.00	1.00	1.00
Female		–	0.75	1.50	0.75	1.39
	–		(0.16–3.56)	(0.51–4.42)	(0.20–2.80)	(0.09–22.59)
Intake of refined carbohydrate in-between-meals three times a day or more						
No		–	1.00	1.00	1.00	–
Yes	–		0.95	4.21	2.94	
			(0.11–8.43)	(1.56–11.37)	(0.69–12.49)	
Dental service utilization in the past 12 months						
No				1.00		
Yes	–	–	–	0.86	–	–
				(0.11–6.97)		
Use of fluoridated toothpaste						
No		1.00	1.00	1.00		
Yes	–	0.08	0.17	0.29	–	–
		(0.004–1.37)	(0.03–01.01)	(0.05–1.54)		
Toothbrushing more than once a day						
No			1.00	1.00	1.00	
Yes	–	–	0.44	3.55	0.11	–
			(0.04–4.28)	(0.28–45.21)	(0.02–0.61)*	
R^2^		0.13	0.06	0.08	0.08	0.02

*p < 0.05

## Discussion

This study produces the first population-level information on the severity of ECC and the prevalence of associated complications in Nigeria. It is also the first study to measure the prevalence of ECC in Nigeria using the ICDAS and SiC index. The results indicate that the overall prevalence of cavitated and non-cavitated carious lesion in the study population was low; there was almost a doubling of the ECC prevalence by age 12–23 months and a four-fold increase (a three-fold increase in cavitated and non-cavitated lesions) by age 24–35-months-old when compared to those age 6–11-months-old. The SiC score was also highest at age 12–23 months, and children 6–37-months old had higher SiC scores than the SiC score for the study population. We also observed that ECC complications occurred at all ages except for those below 1-year-old. The only risk indicator for ECC was toothbrushing, and this was only significantly associated with lower SiC in children age 48–59-months. The teeth worst affected by caries were teeth #85 and #61 (teeth reaching the three topmost dmft, dmfs, and pufa scores).

One of the strengths of the study is that it provides details about age-specific risk indicators for ECC in the study population. It, therefore, allows for planning age-targeted interventions. We found that there are sub-groups of pre-school children with high levels of ECC which require public health response. Though those aged 24–35-months old appear to be at a critical year when the dmft, dmfs peaks, the ECC caries crisis seems to have started evolving by age 12–23-months-old where we find the highest SiC score and a doubling of the caries prevalence. This may indicate that the weaning diets of these children may be high-caries risk diets. There is little known about the weaning diet of the population, but the finding of this study suggests that the sugar content of the diet may be a cause of concern for ECC risk. Though a large population of mothers in the study community breastfed their children up to 24 months [45], the duration of breastfeeding was not identified as a risk indicator for ECC in the study population [29, 42]. A prior study conducted in pre-school children in Lagos, Nigeria indicated, however, that breastfeeding longer than 18 months and exclusive breastfeeding was associated with higher dmft [46].

The study finding indicated that caries prevention practices for children in this population need to start by age 6–11-months when the cariogenic diet is low. This preventive practice should include the use of fluoridated toothpaste and twice-daily toothbrushing though this study did not identify them as risk indicators for the population worst affected by ECC. A prior study conducted in the study environment indicated that the combination of the use of fluoridated toothpaste and twice-daily tooth brushing was the most effective caries prevention measure for children in the study environment with both primary and permanent dentition [47]. ECC prevention messages may be useful in this context and maybe reinforced using multiple possible settings such as the immunization clinics with emphasis on effective tooth brushing more than once a day [48]. These measures can help reduce the increase by age in the prevalence of cavitated and non-cavitated caries and the development of ECC complications observed in this study.

Also, unlike the recommendation made by El Tantawi et al. [49] on the need for epidemiological surveys on ECC to report separately on children 2 years and below versus children 3–5-years-old, we believe that the epidemiological profile of ECC for children 1-year-old and below should be reported distinctly from that of children older than 1-year-olds. We noticed that a difference in ECC profile starts to develop after the age of 1-year indicating the need for ECC prevention programs to be instituted for children in the study population earlier than one year. ECC prevention programs initiated during the antenatal periods were highly successful [50, 51], with those initiated at the age of one year having the greatest impact [50].

In addition, the risk indicators associated with ECC severity differed for each age group. While the intake of refined carbohydrates in-between-meals was associated with a higher dmft score for all age groups except those 60–71-months-old, it was only significantly associated with the dmft score in children 24–35-months-old. The use of fluoridated toothpaste also was associated with lower dmft scores for all age groups and significantly so for children 24–35-months-old. The significant indicator associated with lower dmft in children 48–59-months-old was tooth brushing twice daily or more. Past studies had indicated that consumption of refined carbohydrates and poor oral hygiene were risk indicators for ECC in this study population [28]. What the results of this study suggest is that efforts to control the consumption of refined carbohydrates in-between-meals three times a day or more to reduce the risk of high dmft scores need to start before 24 months. On the other hand, the emphasis on good oral hygiene as a prevention strategy for ECC may be more strategic at an age later than 24–35-months. This study, for the first time, identified that the use of fluoride-containing toothpaste can protect against high dmft in contrast to a past ECC study in the study environment which was not able to find this association [28]. Thus, an age-specific approach for tailoring ECC risk reduction strategies in communities with low ECC prevalence may be effective in planning for the elimination of ECC in these communities.

Also, like a past study [52], we report a high prevalence and higher severity of ECC in the upper central incisors and lower molar teeth. The lower molar teeth seem to be more prone to caries complications with implications for associated pain, negative impact on growth and development [53], and negative impact on the quality of life of both the child and the mother [54]. The importance of this for the developing child is enormous. The impact of caries-associated pain on children’s social interactions and psychological development was previously highlighted [55]. Public health intervention is therefore required in this study population to mitigate the impact of ECC and address its burden in toddlers in this population. These interventions include promoting access of children to oral health care services by their first birthday or as soon as the first tooth erupts [43]. Also, early intervention with pregnant mothers during the perinatal visits may help reduce the risk of ECC [51, 56].

Finally, preventive care for children should include the use of topical fluoride and fissure sealants because the teeth worst affected by ECC are #85 and #61. Topical fluoride is essential to prevent smooth surface caries to which the upper central incisors are predisposed [57]. Fissure sealants are of value in preventing fissure caries for which the primary second molar are predisposed through the evidence of its benefits in preventing or arresting non-cavitated occlusal caries in the primary molars are still not well established [58].

This study has few limitations. Several studies have reported the limitation associated with the use of the dmft index for caries assessment. This includes high inter-observer bias and variability [59]. Our use of the ICDAS helps us to increase the validity of our caries profile as the ICDAS is more sensitive to identifying non-cavitated lesions than the dmft and dmfs [57]. The inter-examiner reliability for this study is also high therefore reducing the risk of examiner variability. In addition, the cross-sectional design of the study cannot support causality and can only suggest associations that need to be further addressed in longitudinal studies. Despite these limitations, the study provides new information and a profile of ECC in a population with low ECC prevalence.

## Conclusion

The study finding suggests that there are age group-specific risk indicators of ECC; and for this study population, tooth brushing once a day or less may be a risk factor for children age 48–59-months. Though the ECC prevalence is low in the study population, the age group with the worst ECC experience was children 24–35-months-old. Interventions to reduce the risk of ECC and its sequelae should begin by 12 months of age at the latest, with the focus being on reinforcing the use of a low cariogenic diet and promoting the use of fluoridated toothpaste. For children 24–35-months-old, the application of topical fluoride and fissure sealants should be included as part of the caries prevention interventions. Oral health policies to push and monitor this oral health care paradigm is needed for the study population.

## Data Availability

Study-related data and materials are accessible on request from the lead author.

## Abbreviations

dmft: Decayed, missing, and filled tooth
dmfs: Decayed, extracted, and filled tooth surfaces
ECC: Early childhood caries
ICDAS: International caries detection and assessment system
pufa: Pulp, ulceration, fistula and abscesses
SiC: Significant caries
